# The SGLT2 Inhibitor Luseogliflozin Rapidly Normalizes Aortic mRNA Levels of Inflammation-Related but Not Lipid-Metabolism-Related Genes and Suppresses Atherosclerosis in Diabetic ApoE KO Mice

**DOI:** 10.3390/ijms18081704

**Published:** 2017-08-04

**Authors:** Yusuke Nakatsu, Hiroki Kokubo, Batmunkh Bumdelger, Masao Yoshizumi, Takeshi Yamamotoya, Yasuka Matsunaga, Koji Ueda, Yuki Inoue, Masa-Ki Inoue, Midori Fujishiro, Akifumi Kushiyama, Hiraku Ono, Hideyuki Sakoda, Tomoichiro Asano

**Affiliations:** 1 Department of Medical Science, Graduate School of Medicine, Hiroshima University, 1-2-3 Kasumi, Minami-ku, Hiroshima 734-8551, Japan; nakatsu@hiroshima-u.ac.jp (Y.N.); ymmty@hiroshima-u.ac.jp (T.Y.); ga54823@ja3.so-net.ne.jp (Y.M.); urouedakouji@yahoo.co.jp (K.U.); m164596@hiroshima-u.ac.jp (Y.I.); b131831@hiroshima-u.ac.jp (M.-K.I.); 2 Department of Cardiovascular Physiology and Medicine, Graduate School of Medicine, Hiroshima University, 1-2-3 Kasumi, Minami-ku, Hiroshima 734-8551, Japan; hkokubo@hiroshima-u.ac.jp (H.K.); bumdelger@hiroshima-u.ac.jp (B.B.); yos1956oktbh@hiroshima-u.ac.jp (M.Y.); 3 Center for Translational Research in Infection & Inflammation, School of Medicine, Tulane University, New Orleans, LA 70118, USA; 4 Division of Diabetes and Metabolic Diseases, Nihon University School of Medicine, Itabashi, Tokyo 173-8610, Japan; fujishiro.midori@nihon-u.ac.jp; 5 Division of Diabetes and Metabolism, The Institute for Adult Diseases, Asahi Life Foundation, Chuo-ku, Tokyo 103-0002, Japan; a-kushiyama@asahi-life.or.jp; 6 Department of Clinical Cell Biology, Chiba University Graduate School of Medicine, 1-8-1 Inohana, Chuo-ku, Chiba-shi, Chiba 260-8670, Japan; hono-tky@umin.ac.jp; 7 Division of Neurology, Respirology, Endocrinology and Metabolism, Department of Internal Medicine, Faculty of Medicine, University of Miyazaki, 5200 Kihara, Kiyotake, Miyazaki 889-1692, Japan; hideyuki_sakoda@med.miyazaki-u.ac.jp

**Keywords:** diabetes mellitus, atherosclerosis, SGLT2 inhibitor, luseogliflozin, hyperlipidemia, inflammation

## Abstract

Recent clinical studies have revealed the treatment of diabetic patients with sodium glucose co-transporter2 (SGLT2) inhibitors to reduce the incidence of cardiovascular events. Using nicotinamide and streptozotocin (NA/STZ) -treated ApoE KO mice, we investigated the effects of short-term (seven days) treatment with the SGLT2 inhibitor luseogliflozin on mRNA levels related to atherosclerosis in the aorta, as well as examining the long-term (six months) effects on atherosclerosis development. Eight-week-old ApoE KO mice were treated with NA/STZ to induce diabetes mellitus, and then divided into two groups, either untreated, or treated with luseogliflozin. Seven days after the initiation of luseogliflozin administration, atherosclerosis-related mRNA levels in the aorta were compared among four groups; i.e., wild type C57/BL6J, native ApoE KO, and NA/STZ-treated ApoE KO mice, with or without luseogliflozin. Short-term luseogliflozin treatment normalized the expression of inflammation-related genes such as F4/80, TNFα, IL-1β, IL-6, ICAM-1, PECAM-1, MMP2 and MMP9 in the NA/STZ-treated ApoE KO mice, which showed marked elevations as compared with untreated ApoE KO mice. In contrast, lipid metabolism-related genes were generally unaffected by luseogliflozin treatment. Furthermore, after six-month treatment with luseogliflozin, in contrast to the severe and widely distributed atherosclerotic changes in the aortas of NA/STZ-treated ApoE KO mice, luseogliflozin treatment markedly attenuated the progression of atherosclerosis, without affecting serum lipid parameters such as high density lipoprotein, low density lipoprotein and triglyceride levels. Given that luseogliflozin normalized the aortic mRNA levels of inflammation-related, but not lipid-related, genes soon after the initiation of treatment, it is not unreasonable to speculate that the anti-atherosclerotic effect of this SGLT2 inhibitor emerges rapidly, possibly via the prevention of inflammation rather than of hyperlipidemia.

## 1. Introduction

It is well established that the development of cardiovascular (CV) disease or atherosclerosis is accelerated in patients with a clustering of risk factors [1,2]. This clustering, known as metabolic syndrome, includes the established risk factors of visceral obesity, hyperlipidemia, hyperglycemia, hyperinsulinemia with insulin resistance, hyperuricemia and hypertension [3,4]. From the standpoint of pathogenesis, inflammation is profoundly involved in the development of atherosclerosis. Indeed, increases in inflammatory cytokines are well known to be closely linked with the exacerbation of atherosclerosis. For example, tumor necrosis factor (TNF) increases intracellular adhesion molecule 1 (ICAM-1) expression, and enables monocytes to differentiate into macrophages which become foam cells via lipid uptake. Increased expression of inflammatory cytokines, which are secreted by macrophages, then leads to the destabilization of plaque in the aorta [5].

Thus, recently, the question of whether administering drugs targeting these disease risk factors actually reduces the incidence of CV events or deaths has received considerable research attention [6,7,8]. In particular, since recent advances in the development of anti-diabetic drugs have provided numerous therapeutic options for patients with type 2 diabetes mellitus, the effects of individual anti-diabetic drugs on the prevention of CV events have been extensively investigated in both basic and clinical studies [9,10].

The SGLT2 inhibitors are a unique class of anti-diabetic drug, of which the mechanism of action is increased urinary glucose excretion, which leads to the lowering of the blood glucose concentration. Although the mechanism of SGLT2 inhibitor action is simple and independent of lipid metabolism, insulin secretion and insulin sensitivity, the above-mentioned CV risk factors are reportedly also improved significantly as secondary effects in association with the normalization of blood glucose concentrations [11,12,13,14,15]. Similarly, recent clinical studies have revealed favorable effects of SGLT2 inhibitors on the prevention of CV events [16,17,18]. The mechanisms of CV event prevention are not fully understood, though diuretic effects and thrifty substrates have been suggested [19,20]. Atherosclerosis is one of the major causes of CV events, including ischemia and heart failure, as well as stroke. In addition, diabetes is closely linked to the development of atherosclerosis, while lipid-lowering treatment also contributes to the prevention of atherosclerosis [21]. Therefore, we investigated whether SGLT2 inhibitor administration would suppress the development of atherosclerosis, and the mechanism of its protective effect.

In this study, we prepared ApoE KO mice treated with both nicotinamide and streptozotocin (NA/STZ) as a diabetic and a hyperlipidemic rodent model exhibiting rapid progression of atherosclerosis. ApoE KO mice are the rodent model most frequently used for studies of atherosclerosis. Considering the background of ApoE KO mice, C57BL/6J mice were used as a normal control. Then, the SGLT2 inhibitor luseogliflozin was administered to the NA/STZ-treated ApoE KO (NA/STZ-ApoE KO) mice.

## 2. Results

### 2.1. The SGLT2 Inhibitor, Luseogliflozin, Suppresses the Expression of Inflammatory Cytokines Induced by Short-Term Hyperglycemia

**Figure 1:**
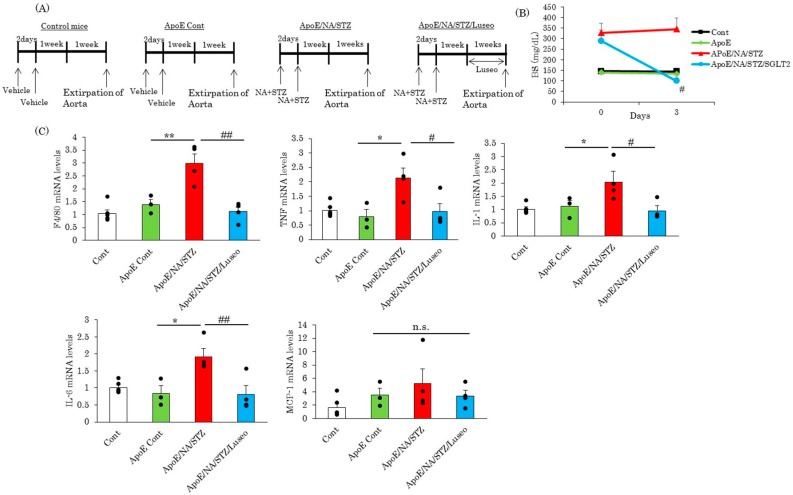
Luseogliflozin abolished upregulation of inflammatory cytokines. (**A**) Scheme of experimental procedure; (**B**) Measurement of blood glucose. Blood glucose levels were measured both before and three days after starting treatment with luseogliflozin; (**C**) Quantitative real-time PCR of inflammatory cytokine genes in the aorta. Whole aortas were extirpated seven days after luseogliflozin treatment and total RNA was extracted. Data are expressed as means + SEM (*n* = 3–6). * *p* < 0.05, ** *p* < 0.01 vs. ApoE cont, ^#^ *p* <0.05, ^##^ *p* < 0.01 vs. ApoE/NA/STZ. n.s.: not significant.

Figure 1A shows the protocols for the injection of NA (120 mg/kg) and STZ (100 mg/kg) to eight-week-old C57BL/6J mice to achieve the development of mild diabetes. One week later, luseogliflozin was given to half of the NA/STZ-ApoE KO mice, while wild type C57BL/6J mice and untreated ApoE KO mice were used as controls. Luseogliflozin was administrated to NA/STZ-ApoE KO mice for seven days and experiments were then performed (Figure 1A). While the serum blood glucose concentrations of NA/STZ-ApoE KO mice were approximately 350 mg/dL, the group receiving luseogliflozin treatment for three days showed normalization of the elevated glucose concentration to around 100 mg/dL (Figure 1B). Two weeks after NA/STZ treatment with or without one-week luseogliflozin administration, no visible atherosclerotic changes were detected in any of the groups, since these mice were still only 10 weeks old. At this pre-atherosclerotic time-point, the alterations of the mRNA levels in the aorta were compared. It was first revealed that a deficiency of the ApoE gene had little impact on the mRNA levels of inflammatory cytokines in the aorta, as shown by the comparison between wild type C57BL/6J and ApoE KO mice. However, a macrophage marker, F4/80, and inflammatory cytokine genes, such as TNF, interleukin (IL)-1 and IL-6, but not MCP-1, were markedly elevated in the NA/STZ-ApoE KO, as compared with the untreated ApoE KO mice. Importantly, administration of luseogliflozin for one week completely removed the elevation of these genes in NA/STZ-ApoE KO, with the same levels as the control ApoE KO mice, the only exception being MCP-1 (Figure 1C).

### 2.2. Luseogliflozin Treatment Normalized the Expression of Adhesion Molecules and Matrix Metalloproteinases, but Had Little Effect on Lipid Metabolism-Related Genes in the Aortas of NA/STZ-ApoE KO Mice

**Figure 2:**
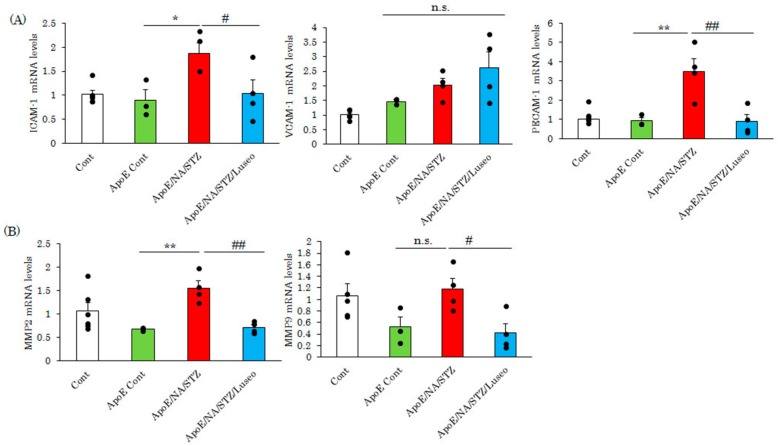
Luseogliflozin decreased the expression of both adhesion molecules and matrix metalloproteinases. (**A**,**B**) Real-time PCR of adhesion molecules or matrix metalloproteinases in the aorta. Whole aortas were extirpated seven days after luseogliflozin treatment and total RNA was extracted. Data are expressed as means + SEM (*n* = 3–6). * *p* < 0.05, ** *p* < 0.01 vs. ApoE cont ^#^ *p* < 0.05, ^##^ *p* < 0.01 vs. ApoE/NA/STZ. n.s.: not significant.

The attachment of macrophages to the aortic wall is a first step in plaque formation, and several adhesion molecules, such as ICAM-1, play a critical role in this process. In the aortas of NA/STZ-ApoE KO mice, the expression of adhesion molecule genes such as ICAM-1 and the platelet endothelial cell adhesion molecule 1 (PECAM-1) were increased as compared with control ApoE KO mice (Figure 2A), while no significant differences were observed between wild type C57BL/6J and ApoE KO mice at the age of 10 weeks. In addition, elevated expressions of ICAM-1 and PECAM-1 in the aortas of NA/STZ-ApoE KO mice were normalized to the levels of wild type C57BL/6J and ApoE KO mice (Figure 2A).

Similarly, the expression of matrix metalloproteinase-2 and -9, which are involved in plaque destabilization, were also elevated in the NA/STZ-ApoE KO mice and normalized in response to the one-week treatment with luseogliflozin (Figure 2B).

**Figure 3:**
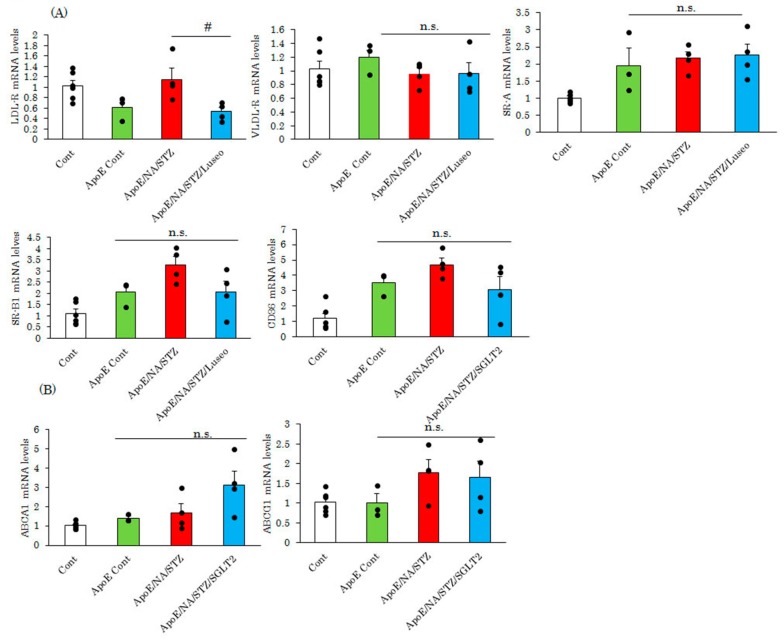
Luseogliflozin exerted minimal effects on the expression of lipid metabolism-related genes. (**A**,**B**) Expression levels of scavenger receptors and cholesterol efflux transporters in the aorta were measured by real-time PCR. Whole aortas were extirpated seven days after luseogliflozin treatment and total RNA was extracted. Data are expressed as means + SEM (*n* = 3–6). ^#^ *p* < 0.05 vs ApoE/NA/STZ. n.s.: not significant.

After infiltrating the aorta, macrophages undergo foaming which is a critical event for atherosclerosis development. In this step, lipids, particularly oxidized or denatured LDLs, are incorporated via scavenger receptors. Therefore, we investigated whether the SGLT2 inhibitor changed the mRNA levels of scavenger receptors. At the age of 10 weeks, the mRNA expression of SR-A, SR-B1 and CD36 were elevated in ApoE KO mice, as compared with wild type C57BL/6J. However, interestingly, neither NA/STZ nor luseogliflozin treatments significantly affected scavenger receptor expression, with the only exceptions being the upregulation and normalization of LDL-R mRNA expression by NA/STZ and luseogliflozin treatments, respectively (Figure 3A). ABCA1 and ABCG1 both have the ability to export cholesterol. Although expressions of these genes did not differ significantly among wild type C57BL/6J, untreated and NA/STZ-treated ApoE KO mice, luseogliflozin treatment tended to increase the expression of only ABCA1, i.e., not that of ABCG1(Figure 3B).

### 2.3. Luseogliflozin-Treatment Had No Effect on the Hyperlipidemia in ApoE KO Mice

**Figure 4:**
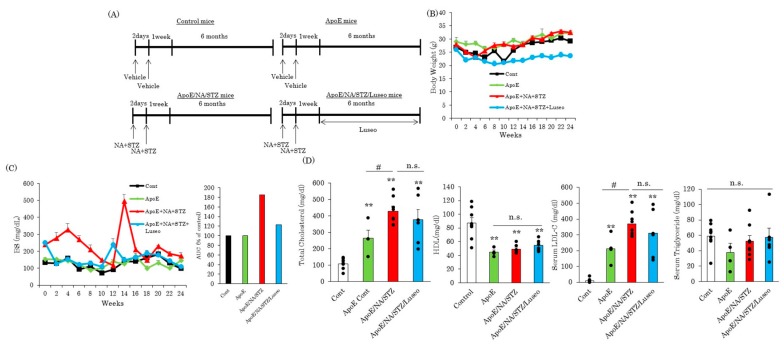
Luseogliflozin exerted no influences on serum parameters. (**A**) Scheme of experimental procedure; (**B**) Changes in body weight; (**C**) Changes in blood glucose levels; (**D**) Serum parameters. Serum was collected six months after treatment with or without luseogliflozin. Data are expressed as means + SEM (*n* = 4–8). ** *p* < 0.01 vs. Cont ^#^ *p* < 0.05 vs. ApoE Cont. n.s.: not significant.

To investigate the effects of the SGLT2 inhibitor luseogliflozin on atherosclerosis development, this drug was continuously administered to NA/STZ-treated ApoE KO mice for six months (Figure 4A). While the body weights of wild type C57BL/6J, untreated and NA/STZ-treated ApoE KO mice were similar, luseogliflozin treatment significantly suppressed weight gain in NA/STZ-treated ApoE KO mice with the normalization of hyperglycemia (Figure 4B,C). As expected, wild type ApoE KO mice showed markedly higher levels of serum cholesterol and LDL, while serum HDL levels were lower, as compared to wild type C57BL/6J mice (Figure 4D). Serum triglyceride levels did not differ between control mice and wild type ApoE KO mice. Interestingly, these serum lipid parameters were unaffected by luseogliflozin administration to NA/STZ-treated ApoE KO mice (Figure 4D), although the NA/STZ-induced diabetic state was associated with more severe hyperlipidemia.

### 2.4. Hyperglycemia Remarkably Exacerbates Atherosclerosis in ApoE KO Mice, but Luseogliflozin Prevents the Worsening of This Atherosclerosis

**Figure 5:**
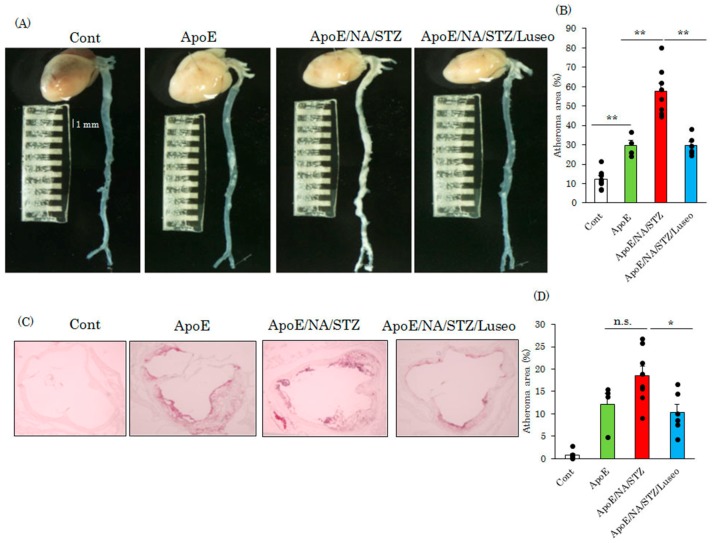
Luseogliflozin markedly suppressed the development of atherosclerosis. (**A**) Representative photographs of whole aorta. Aortas were extirpated six months after treatment with or without luseogliflozin; (**B**) Quantitative data for plaque area. Data are expressed as means + SEM (*n* = 4–8); (**C**) Oil red O staining of aortic root areas; (**D**) Quantitative data for oil red O positive area. * *p* < 0.05, ** *p* < 0.01 vs. ApoE cont. n.s.: not significant.

Finally, the areas of the atherosclerotic regions were compared among the groups of wild type C57BL/6J, untreated ApoE KO and NA/STZ-treated ApoE KO mice with or without luseogliflozin administration. Figure 5A shows the unstained heart and whole aorta specimens of these four groups of mice. While the normal portion of the aorta is thin and thus appears transparent, regions showing severe progression of atherosclerotic lesions are thickened and thus appear whitish (Figure 5A). The percentage of the whitish portion was calculated and is shown in Figure 5B. At the age of approximately eight months, no plaque was observed in the wild type C57BL/6J mice, but atherosclerotic areas were readily detectable in the untreated ApoE KO mice. While the atheroma area occupied approximately 30% of the aorta in untreated ApoE KO mice, this area accounted for almost 60% in that of NA/STZ-ApoE KO mice. In addition, luseogliflozin administration to NA/STZ-ApoE KO mice reduced the atheroma area to a level of 30%, similar to that in the non-diabetic ApoE KO mice (Figure 5A,B). These data were also supported by the observation by Oil Red O staining of the aortic root areas, which were reduced by luseogliflozin to the levels seen in untreated ApoE KO mice (Figure 5C,D).

**Figure 6:**
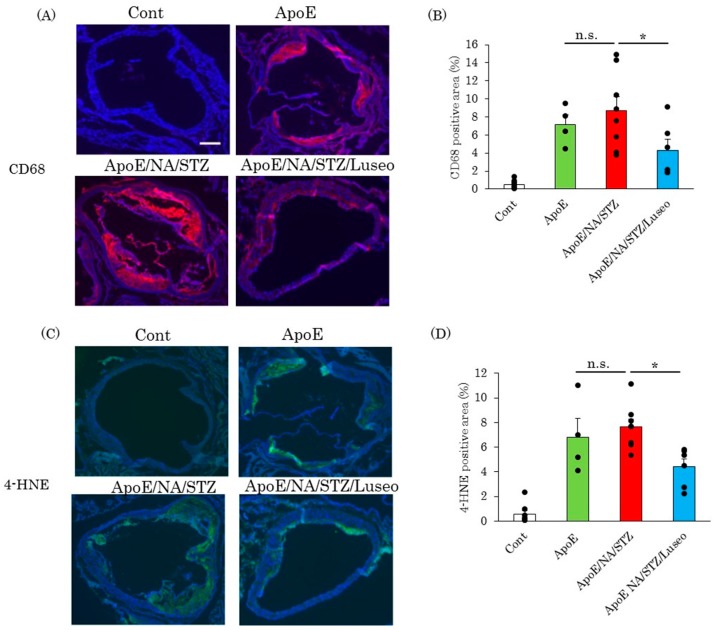
Luseogliflozin inhibited macrophage infiltration and lipid peroxidation. (**A**,**B**) Representative photographs of CD68 staining in aortic root areas and quantitative data are shown; (**C**,**D**) Slides of aortic tissue were stained with 4-HNE antibodies. Representative photographs and quantitative data are shown. Aortas were extirpated six months after treatment with or without luseogliflozin. Data are expressed as means + SEM (*n* = 4–8). Scale bar = 200 μm.

As the diabetic state results in the upregulation of the expression of F4/80, a macrophage marker (Figure 1C), we investigated macrophage infiltration of the aortic root area. CD68 staining revealed that macrophage infiltration is ameliorated by luseogliflozin (Figure 6A,B). Similarly, staining for 4-hydroxynonenal, a marker of lipid peroxidation, was widely observed in the aortas of NA/STZ-treated ApoE KO mice and the group given luseogliflozin showed smaller areas of staining (Figure 6C,D). In general, the degrees of macrophage infiltration and oxidative stress accumulation detected by immunostaining with anti-CD68 and anti-HNE antibodies, respectively, correlated well with the sizes of the atheroma areas.

## 3. Discussion

Recently, numerous anti-diabetic drugs with diverse mechanisms of action have become available. Faced with the challenge of choosing the optimal drug for treating diabetic patients, not only potency for lowering the blood glucose concentration but also long-term effects on the incidence of various events including death have been regarded as important factors [16,22]. First, drugs which reduce or at least do not affect body weight tend to be preferred to those exacerbating obesity. Based on this known property, the uses of three types of anti-diabetic drugs, specifically biguanides, incretin or its related medications such as DPP4 inhibitors, and SGLT2 inhibitors have been increasing. Furthermore, another matter of major interest has been whether or not each of these types of anti-diabetic drugs, particularly incretin-related medications and SGLT2 inhibitors, exert a reducing effect on the incidences of CV events and the mortality rate [16,22,23,24].

SGLT2 inhibitors simply increase the urinary secretion of glucose and thereby lower the blood glucose concentration. In this study, we used NA/STZ-ApoE KO mice as a hyperglycemic and hyperlipidemic rodent model [25], fed normal chow to avoid the development of obesity-related insulin resistance. The elevated glucose concentration in the NA/STZ-ApoE KO mice was normalized by the administration of luseogliflozin, and this normalization took no longer than three days. A gradual decrease in blood glucose levels was observed during long term-experiments. However, we have been unable to elucidate why blood glucose levels showed changes. One possibility is that the regeneration of pancreatic β cells may take place during experimental periods. Indeed, the proliferation of β cells was reportedly observed after the injection of low-dose STZ [26].

At this time point during short-term administration, no obvious atherosclerotic lesions [27] were detected by visual or microscopic observation, possibly due to the young age of the mice (10 weeks old). However, the expression of inflammation-related genes, involved in atherosclerosis development [28,29,30,31,32,33], were markedly elevated in the aortas of NA/STZ-ApoE KO mice, as compared with untreated ApoE KO mice. The inflammation-related genes included F4/80, TNFα, IL-1β, IL-6, ICAM-1, PECAM-1, matrix metalloproteinase MMP2 and MMP9, and interestingly, these increased mRNA levels were normalized by luseogliflozin treatment to the levels in normal ApoE KO mice, despite the short period (seven days) of administration. Thus, it was suggested that inflammation in the aorta is very likely to be intense and to occur rapidly in the presence of hyperglycemia. Previous reports have demonstrated that TNFα or IL-1β knockout mice show an alleviation of atherosclerosis, without an improvement in serum lipid parameters [29,34].

Regarding the molecular mechanisms possibly linking hyperglycemia with cytokine expression, reactive oxygen species (ROS) accumulation appears to be the most likely candidate [35]. Indeed, the production of ROS and advanced glycation end products and the associated receptors [36,37], which are increased by hyperglycemia, induce systemic inflammation and the progression of diabetic complications [38], as well as impairing endothelial function and accelerating atherosclerosis development [39,40].

In fact, recent reports have shown the suppression of arteriosclerosis or endothelial dysfunction in response to SGLT2 inhibitor administration [41,42,43]. In these prior studies, the effects of long-term SGLT2 inhibitor administration on inflammation and insulin resistance, or ROS and inflammasomes, were investigated. In addition, their hyperglycemic models showed insulin resistance induced by a western diet, and thus, the lipid profiles were also improved by long-term treatment with SGLT2 inhibitors. In contrast, in our study, most of the examined lipid metabolism-related genes, such as VLDL-R, SR-A, SR-B1, CD36, and ABCG1, were unaffected by either NA/STZ-induced hyperglycemia or treatment with luseogliflozin, although the reduction in LDL-R and the increase in ABCA1 mRNAs were exceptions, observed with seven days of luseogliflozin treatment.

In our study, at the six-month endpoint after NA/STZ followed by luseogliflozin treatment, a marked difference in the degree of atherosclerosis was observed. These results clearly indicate that hyperglycemia alone, not necessarily accompanied by insulin resistance, is sufficient for atherosclerosis acceleration in the presence of hyperlipidemia. It should be noted that no significant differences were observed in the serum lipid parameters among the untreated ApoE KO, NA/STZ-ApoE KO and luseogliflozin-treated NA/STZ-ApoE KO groups of mice.

## 4. Materials and Methods

### 4.1. Animals, Diets and Luseogliflozin Treatment

To induce mild to moderate diabetes in C57BL/6J mice, NA (120 mg/kg) and then STZ (100 mg/kg) was injected after starvation for 20 h and the mice were then given a normal chow diet (ND) (Oriental Yeast, Tokyo, Japan), as shown in Figure 1A. After one week, mice showing mild diabetes were randomly divided into two groups. The SGLT2 inhibitor luseogliflozin (TS-071: (1*S*)-1,5-anhydro-1-[5-(4-ethoxybenzyl)-2-methoxy-4-methylphenyl]-1-thio-d-glucitol), synthesized by Taisho Pharmaceutical Co., Ltd.(Tokyo, Japan) [44], was given to one of the two groups, by mixing it into the diet at a concentration of 0.1%, to normalize mild hyperglycemia [25]. This dosage of luseogliflozin given with the food was selected to assure the maximal glucose-lowering efficacy, even in the event of differences in daily food intakes among mice or in an individual mouse. Then, mice were bred while receiving a normal diet for one week or six months, followed by the extraction of the aorta. As a control, we used C57BL/6J mice, and native ApoE KO mice were also included in the experimental groups. All groups were allowed free access to water and a normal diet for the experimental period. This experiment was approved by the animal committee of Hiroshima University (approval number: A13-26, 15 May 2013).

### 4.2. Evaluation of Atherosclerotic Area and Histochemical Studies

Aortas were removed from mice with the hearts attached. The aortic cavities were opened. While the normal portion of the aortic wall was thin and appeared transparent, the portions with severely advanced atherosclerosis were observed to be whitish due to thickening (Figure 5A). These alterations were evaluated by visual observation, and the ratios of atherosclerotic areas per whole wall area were quantitatively analyzed for comparison among groups. In addition, frozen sections of aortic roots were prepared for Oil Red O staining and immunohistochemistry. For Oil Red O staining to examine triglyceride accumulation, the sections were frozen in liquid nitrogen and embedded in OTC (optimum cutting temperature) compound. After staining, these sections were washed and embedded. For immunostaining, sections were incubated in 0.1% Triton for 5 min to achieve permeabilization, and then boiled in citrate buffer for antigen activation. After being washed with phosphate buffered saline (PBS), the sections were reacted with CD68 or 4-HNE antibodies (1:400) at 4 °C overnight, and Cy3 or Fluorescein-conjugated secondary antibody was then applied to the slides. Finally, the slides were mounted with 4′,6-diamidino-2-phenylindole (DAPI).

### 4.3. Measurements of Serum Parameters

Serum triglyceride, total cholesterol and HDL were measured with the Triglyceride E-test (Wako, Osaka, Japan), cholesterol E-test (Wako) and HDL E-test (Wako), respectively. Serum LDL was calculated employing the Friedewald equation (LDL = TC − HFDL − TG/5).

### 4.4. Quantitative Real Time Reverse Transcription PCR

Mice were anesthetized and the heart and whole aorta were both extirpated, after blood collection. Extirpated tissues were rapidly frozen in liquid nitrogen. Then, total RNA was extracted from the whole aorta using Sepasol reagent (Nakalai Tesche, Kyoto, Japan). Template cDNA was obtained using total RNA employing a Verso cDNA synthesis kit (Thermo Scientific, Waltham, MA, USA), and quantitative real time PCR was carried out using SYBR Green PCR master mix (Invitrogen, Tokyo, Japan). Each expression level was normalized by actin levels, which had been measured as an internal control. The designed primers were as follows; F4/80 F: tctggggagcttacgatgg R: taggaatcccgcaatgatgg, TNF F: gccaccacgctcttctgtc R: tgctcctccacttggtggtt, IL-1β F: ttgacggaccccaaaagatg R: agctgccacagcttctccac, IL-6 F: ccatccagttgccttcttgg R: tgcaagtgcatcatcgttgt MCP-1 F: aggtccctgtcatgcttctg R: tctggacccattccttcttg, ICAM-1 F: gtctcggaagggagccaag R: tgcagttccagggtctggtt VCAM1 F: tcttgggagcctcaacggta R: tgacaggctccatggtcaga, CD36 F: gagcaactggtggatggttt R: gcagaatcaagggagagcac, LDL-R F: cccacatctgcaaggacctc R: ctatggagcccacagccttg, VLDL-R F: acaaatggccgctgcattac R: gaaccgtcttcgcaatcagg, ABCA1 F: gaacagctccagctcctcca R: tccacgtcttcctcggtgtt, ABCG1 F: cctcacccagttctgcatcc R: agggcagcaaacatgaggaa, SR-A1 F: ccttcattcaagggcctcct R: ctcctgggtttcctcgacct, SR-B1 F: caagcagcaggtgctcaaga R: ggttctccacgaaggacacg, MMP9 F: aacctccaacctcacggaca R: tcccacttgaggcctttgaa, MMP2 F: acacctgacctggaccctga R: tgtgccaggagtccatcctt, PECAM1 F: gcacttagaccaggccatgc R: tccccagtggaaggagtgaa.

### 4.5. Statistical Analysis

Results are expressed as means ± SEM. Statistical significance was assessed using ANOVA followed by the Tukey HSD test. Values of *p* < 0.05 were taken to indicate a statistically significant difference. Statistical analysis was performed, using Ekuseru-Toukei 2015 (Social Survey Research Information Co., Ltd., Osaka, Japan).

## 5. Conclusions

Taken together, our data suggest that hyperglycemia rapidly induces an inflammatory state in the aorta, which is normalized by short-term (seven days) treatment with the SGLT2 inhibitor luseogliflozin. Although further studies are necessary to fully elucidate the molecular mechanism underlying aortic inflammation induced by hyperglycemia, the anti-atherosclerotic effects of SGLT2 inhibitors might be mainly attributable to suppressed inflammation, which would eventually contribute to the prevention of CV events in diabetic patients.
